# Psychometric Analysis of the JSPE Nursing Student Version R: Comparison of Senior BSN Students and Medical Students Attitudes toward Empathy in Patient Care

**DOI:** 10.5402/2011/726063

**Published:** 2011-05-11

**Authors:** Libba Reed McMillan, David M. Shannon

**Affiliations:** School of Nursing, Auburn University, 221 Miller Hall, Auburn, Al 36839, USA

## Abstract

*Background*. Empathic communication skills are critical to providing high-quality nursing care to holistically understand the patient's perspective. A survey research design was used to address the research questions discussed in this study. Data consisted of responses from nursing students attending accredited programs in the southeastern United Sates using the Jefferson Scale of Physician Empathy Nursing Student Version R (JSPE-R). *Findings*. Comparisons of the total scores from JSPE Versions S and R yielded similar means and standard deviations with 115 and 114.57, respectively, and standard deviations of 10 and 10.94, respectively. The results of a one-sample *t*-test failed to render statistical significance (*t* = −1.22, *P* = .224), indicating that the overall attitudes of nursing students and medical students are similar. The 25th, 50th, and 75th percentiles and overall instrument reliability were also comparable. *Conclusions*. This paper supports the emergence of alternative factor analysis structures as applied to nursing students through statistical progression from exploratory factor analysis to confirmatory structures. Implications for practice explore the utility of empathy instruments in nurse education, such as empathy progression through curriculum. As nursing educators, the utility of development of instruments to measure effectiveness of teaching strategies and pedagogy for empathy enhancement in practice is important.

## 1. Introduction

The rationale for analysis of the instrument was to study the psychometric aspects of the Jefferson Scale of Physician Empathy Version R. This instrument was designed to measure nursing student attitudes toward empathy in the patient care setting. At the time of data collection, this instrument was developed as a modification of other versions using a population of health care professionals by researchers affiliated with Thomas Jefferson University Hospital and Jefferson Medical College in the Philadelphia area. The operational definition of the concept of empathy used in the instrument was “empathy is a predominantly cognitive (rather than emotional) attribute that involves an understanding (rather than a feeling) of experiences, concerns, and perspectives of the patient, combined with a capacity to communicate this understanding” [1, page 80]. 

## 2. Background

Critical review of existing literature regarding empathy encompasses works of all aspects of the health care profession, such as nursing, medical, pharmacy, and physical therapy. The concept of empathy is one marked with much misunderstanding, controversy, and confusion. Researchers debate whether empathy is cognitively or emotionally based. Even more debate exists as to whether empathy can be taught to health care professionals. Existing instruments include the Empathy Construct Rating Scale (LaMonica), the Interpersonal Reactivity Index (Davis), the Layton Empathy Test (Layton), and the Jefferson Scale of Physician Empathy [2].

The international relevance of the instrument is important to theory development and to advancement of understanding and measuring the concept of empathy in patient care. Through this understanding, there can be improved methods of instruction to students and improvement of patient care. Empathy has been linked with improved patient outcome measures, and is regarded to be a key determinant of patient and family satisfaction, improved clinical outcomes in the form of recovery and healing, fewer malpractice suits and litigations, and overall positive perspectives of care [3, 4]. Much has been learned about the role of empathy in patient care [5–15] and patient outcomes [3, 4, 16–18].

Empathic communication skills are critical to providing high-quality nursing care to patients in an attempt to holistically understand the patient's perspective. These skills pertaining to therapeutic communication must include evidence that the student: (a) demonstrate communication skills during assessment, intervention, evaluation, and teaching, (b) adapt communication methods to patients with special needs, such as psychological or sensory disabilities, and (c) use therapeutic communication within the nurse-patient relationship, and elicit and clarify patient preference and values (Commission on Collegiate Nursing Education (2009). Standards for accreditation of baccalaureate and Graduate degree Nursing Programs. Retrieved from http://www.aacn.nche.edu/accreditation/pdf/standards09.pdf). This clarification process of patient preferences and values involves the ability of the nurse to understand the patient's perspective and communicate this understanding, both of which involve empathic ability.

In terms of patient outcome measures, effective empathic communication is widely regarded to be a key determinant of patient and family satisfaction, by showing and providing understanding, comfort and support [4, 14, 19–21]. Improved clinical outcomes have been linked with empathic care, such as better recovery, improved healing, fewer malpractice suits, and litigations [22], and an overall positive perspective of patient care [3, 4, 18, 23]. Reynolds and Scott [18] posit that empathy is crucial to the fundamental aim and achievement of nursing goals. These findings indicated that empathy (a) enabled nurses to create a climate of trust and to establish their client's perceptions of need, (b) enabled nurses to judge the client's state and readiness to talk, (c) is needed in order that nurses can understand the origins and purposes of client's responses to health problems, and (d) facilitated positive health outcomes for clients, among which are reduction of anxiety, depression, and physiological distress. The achievement of outcomes is dependent upon the ability of the nurse to offer high levels of empathy to clients (page 231). 

### 2.1. Measurement of Empathy

An important step towards the advancement of empathy as a concept is through empirical contributions that measure empathy. In recognition of the importance of empathy within the healthcare profession, the generic version of the Jefferson Scale of Physician Empathy (JSPE) was developed to examine attitudes of medical students, practicing physicians and other health care professionals [24–28]. Numerous research efforts by Hojat et al. [1, 2] has discovered relationships between total scores of the JSPE and correlated subscale scores of the Interpersonal Reactivity Index (IRI) that were relevant to patient care (empathic concern, and perspective taking). Additionally, personality facets from the NEO PI-R (warmth, dutifulness, and faith-in-people), items from self-report (compassion and sympathy), self-reported personal attributes (empathy, compassion, trust, sympathy, tolerance, personal growth, communication, self protection, humor, and clinical neutrality) were utilized to examine the criterion-related validity of the generic version of the JSPE.

 The JSPE has been modified and applied to various groups within the healthcare profession. These modifications include (a) the HP version, which is applicable to physicians and other health professionals, and (b) the S-Version, which is applicable to students in medical and other health professions. This study was aimed at use of the JSPE for use with nursing students, which is the R-version.

## 3. Aims

The primary purpose of this study was to examine the psychometric properties of Jefferson Scale of Physician Empathy-Nursing Student Version R (JSPE-R). The JSPE-R is a modification of a previous version, the JSPE-S Version, which is designed for use in medical school students. The psychometric properties of the JSPE-R were examined through internal consistency analysis and factor analysis and compared with those obtained from the prior version (JSPE-S) used with medical students. 

In order to investigate the psychometric properties of the JSPE-S, the following research questions were examined.

1. Is there a difference between the psychometric properties of JSPE Version S (developed and used previously with medical students) and a modified version (JSPE Nursing Student Version R) used with nursing students in the current study?
2. Does the three-factor model established by Hojat [1] with physicians and medical students apply to nursing students using the JPSE Nursing Student Version R?
3. If the three-factor model established by Hojat [1] does not yield an acceptable fit, what alternative factor structures emerge from the JSPE Nursing Student Version R scores when applied to a sample of nursing students?

## 4. Methodology

The JSPE Nursing Student Version-R is a modification of the generic version of the Jefferson Scale of Physician Empathy. It is a self-administered survey, containing 20 items, developed by researchers at the Center for Research in Medical Education and Health Care at Jefferson Medical College. The JSPE has been modified for use with various health professionals. The current study utilized the JSPE-R Version for the nursing profession. Consistent with Hojat's prior revisions, the JSPE-S-Version was modified by Hojat for use in the current study by substituting the words “nurses” or “nursing care” instead of “physician” or “medical care”.

## 5. Participants

The population in the study consisted of baccalaureate nursing school seniors attending Commission on Collegiate Nursing Education (CCNE) accredited programs. CCNE accredited programs were selected because the programs had demonstrated successful completion of similar formative and summative evaluation. Data collection was from August 2006 to December 2006.

The final sample consisted of 598 nursing school seniors from 14 nursing programs, an overall mean response rate of 83%. Reponses from the individual programs ranged from 55% to 94% response rates, with a median of 86%. The greatest percentage of participants was female (88%) and Caucasian (83%). Nearly three-fourths of the sample was also under the age of 25 as 46% reported an age between 20–22 and 26% reported their age between ages 23–25. Generalizing to other populations is important to establishing external validity. External validity was supported by comparing the participants to national data. Gender and ethnicity is reported for the current sample and nationally in Table 1.

## 6. Instrumentation

Each participant completed the JSPE Nursing Student Version-R, a modification of the generic version of the Jefferson Scale of Physician Empathy. The instrument is a self-administered survey containing 20 items. The current version maintains the original 7-point response scale (1 = Strongly Disagree to 7 = Strongly Agree). Nine of the 20 items are worded negatively and are reverse coded so that a higher value is attached to a more positive attitude. The items are summed to arrive at a total score with a possible range from 20 to 140.

## 7. Data Analysis

The research questions in this study were addressed using a one-sample *t*-test, exploratory, and confirmatory factor analyses [29]. A one-sample *t*-test was used to compare the current sample of nursing students with the information provided by Hojat [1] for medical students. Second, a confirmatory factor analysis was used to test the applicability of the three-factor model, established by Hojat [1] with the original JSPE-HP Version, to the JSPE-R, designed for use with nursing students in the current study. Specifically, the data were examined using AMOS version (6.0) maximum likelihood factor analysis [30]. The results were evaluated using several criteria. First, departure of the data from the specified model was tested for significance by using a chi-square test [31]. Second, goodness-of-fit between the data and the specified model was estimated by employing the Comparative Fit Index (CFI) [32, 33], the Tucker-Lewis Index (TLI) [34], and the Root Mean Square Error of Approximation (RMSEA) [35]. Based on a recent review of research pertaining to confirmatory factor analysis, Schreiber et al. [36] recommended that the TLI and CFI be >95 for an acceptable fit of the model and a RMSEA <  .06. Earlier reviews [37, 38] have suggested a combination of CFI >  .90 and RMSEA <  .06.

Finally, exploratory factor analysis was used to explore alternative factor structures that may represent the empathy attitudes of nursing students. A Kaiser-Meyer-Olkin Measure (KMO) of Sampling Adequacy of  .829 and statistically significant (*P* < .001) Bartlet's test of sphericity supported the appropriateness of the data for EFA. Specifically, the EFA was performed using a principal component extraction method, and a varimax rotation of 20 self-report JSPE Nursing Student Version R empathy items was conducted on the sample of nursing school seniors (*n* = 598). Factors were initially extracted based on Kaiser's criterion that eigenvalues are larger than 1 [39] and the examination of the resulting scree plot.

## 8. Results

### 8.1. Research Question 1

The first research question was, “Is there a statistical difference between the JSPE Version S and the JSPE Nursing Student Version R? These psychometric properties were examined for the JSPE-Version S using a sample of 685 first year students from three groups of medical students (matriculates of 2002, 2003, and 2004) and compared with these same properties of the JSPE Nursing Student Version R from the current sample of 598 nursing students. These data are summarized in Table 2.

Comparisons of the total scores from JSPE Versions S and R yielded similar means and standard deviations with 115 and 114.57, respectively, and standard deviations of 10 and 10.94, respectively. The results of a one-sample *t*-test failed to render statistical significance (*t* = −1.22, *P* = .224), indicating that the overall attitudes of nursing students and medical students are similar. The 25th, 50th, and 75th percentiles and overall instrument reliability were also comparable.

### 8.2. Research Question 2

The second research question was aimed at testing the hypothesis that the three-factor model established with the JSPE-HP Version by Hojat [1] and applied to medical students would apply to the revised version (JSPE Nursing Student Version R) used with nursing students in the current study. These results from this analysis are summarized in Table 3.

These results offer minimal support for the three-factor model established with the JSPE Version S, resulting in a statistically significant chi-square of 458.79 (*P* < .001). Both the TLI (.821) and CFI (.854) failed to meet the criterion of either  .90 or  .95, recommended for accepting the model. The RMSEA of  .054, however, did meet the criterion of less than  .06.

### 8.3. Research Question 3

Based on the mixed support for Hojat's three-factor model, an alternative factor structure was explored using exploratory factor analysis (EFA). An initial five factor solution emerged. Two of the factors, however, minimally met Kaiser's criterion with eigenvalues of 1.1 and 1.04, so a three factor solution was specified. 

The results of the alternative three-factor solution, summarized in Table 4 are very similar to that of Hojat's findings. These three factors accounted for 38.5% of the total variance compared to 36% for the three-factor structure found by Hojat [1] with medical students. With the exception of three items, the generated standardized factor loadings were higher in the current sample of nursing students. Coefficients less than  .40 were eliminated from consideration, as they were not strongly related to the factor [40–42]. A major difference in Hojat's primary factor is perspective taking while the primary factor in the current study is compassionate care (emotional engagement). Perspective taking emerged as the second factor in the current study. The third factor, “standing in the patient's shoes,” was also comparable to that produced with medical students.

## 9. Discussion

There was no statistical difference in student orientations toward empathy in patient care between medical and nursing students. Simply stated, there are similar responses from nursing and medical students regarding their attitudes to empathy in patient care. From a practical standpoint, there are potential opportunities for collaborative efforts between nursing and medical school programs to share resources of clinical experiences, scenario development, research, and faculty expertise. As members of the health care team, nursing students and medical school students understanding of their commonalities in attitudes toward empathy in patient care should be important to improved relationship and communication among them as team members in the mutual goal of improved patient care. In larger institutions that have both medical and nursing school programs, there are fertile opportunities to expand upon “lessons learned” from caring for patient and or family situations that require advanced skill in communicating empathically, such as the angry patient, dying patients, patients with recent chronic diagnosis, psychiatric conditions and others. 

These findings reinforce the importance of identifying and measuring constructs associated with empathy. Surveys that measure attitudes toward empathy in patient care assist with future research in this important area to patient care. As nursing educators, innovative methods of teaching need to be pursued that delve into understanding perspectives of patients and families. Existing methods of teaching communication and caring should be evaluated for effectiveness in these areas. Dialogue and collaboration with other health care profession educators, inclusive of medical school educators, should be explored. 

Our findings can be further supportive through additional studies to screen for the best items for inclusion and modification in the Nursing Student Version-R for use in nursing student populations. Continued efforts should be made to gather information about empathy and support of the psychometrics of the JSPE Nursing Student Version R. Refinement of psychometrically sound tools are necessary to examine students' attitudes at various stages in the student's program (prenursing, junior level, senior level) and into their career progression as professional nurse. This assists with determination of program's effectiveness in producing positive or negative changes in attitudes toward empathy in patient care.

## Figures and Tables

**Table 1:** Descriptive summary of participants, *comparison of sample group with national population*.

	AACN	Sample
Gender		
Female	90.3%	88%
Male	9.7%	12%
Ethnicity		
African american	12.1%	9%
Asian	5.8%	3%
Caucasian	76.1%	83%
Hispanic	5.2%	2%
Native american	0.8%	0.7%
Other	—	1.7%

AACN demographic figures regarding gender (*n* = 161, 787 students) and ethnicity (*n* = 148, 944 students) enrolled Fall 2005 [43].

**Table 2:** Comparison of JSPE-Version S (*n* = 685 medical students) and JSPE nursing student Version R (*n* = 598 senior nursing students).

JSPE S-VERSION [1] (*n* = 685 medical students)	JSPE R-VERSION (*n* = 598 nursing students)
Mean—115	Mean—114.57
Standard deviation 10	Standard deviation—10.94
25th percentile—108	25th percentile—108
50th percentile—115	50th percentile—116
75th percentile—122	75th percentile—122
Range—75–140	Range—56–140
Cronbach's Alpha—0.80	Cronbach's Alpha—0.77

**Table 3:** Confirmatory factor analysis—Hojat's model.

Model	*χ* ^2^	df	CFI	TLI	RMSEA (90% CI)
Three factors	458.79***	167	.857	.821	.054 (.048 to.060)

**Table 4:** Standardized factor loadings for attitudes toward empathy in patient care.

Item	Hojat [1]^a^			Current study^b^		
	F1	F2	F3	F1	F2	F3
2 Patients feel better when their nurses understand their feelings	.458				.512	

4 Understanding body language is as important as verbal communication in nurse-patient relationships	.480				.424	

5 A nurses's sense of humor contributes to a better clinical outcome					.443	

9 Nurses should try to stand in their patients' shoes when providing care to them	.515				.698	

10 Patients value a nurse's understanding of their feelings which is therapeutic in its own right	.615				.630	

13 Nurses should try to understand what is going on in their patients' minds by paying attention to their nonverbal cues and body language	.510			.509		

15 Empathy is a therapeutic skill without which the nurse's success is limited	.421				.427	

16. Nurses' understanding of the emotional status of their patients, as well as that of their families is one important component of the nurse-patient relationship	.641			.547		

17 Nurses should try to think like their patients in order to render better care					.611	

20 I believe that empathy is an important therapeutic factor in medical treatment	.534			.403		

19x I do not enjoy reading nonmedical literature or the arts (reversed)						

18x Nurses should not allow themselves to be influenced by strong personal bonds between their patients and their family members (reversed)						.542

14x I believe that emotion has no place in the treatment of medical illness (reversed)		.607		.650		

12x Asking patients about what is happening in their personal lives is not helpful in understanding their physical complaints (reversed)		.500		.590		

11x Patients' illnesses can be cured only by medical or surgical treatment; therefore, nurses' emotional ties with their patients do not have a significant influence in medical or surgical treatment (reversed)		.596		.641		

8x Attentiveness to patients' personal experiences does not influence treatment outcomes (reversed)		.604		.608		

7x Attention to patients' emotions is not important in history taking (reversed)		.526		.665		

1x Nurses' understanding of their patients' feelings and the feelings of their patients' families does not influence medical or surgical treatment		.446		.444		

6x Because people are different, it is difficult to see things from patients' perspectives (reversed)			.711			.759

3x It is difficult for a nurse to view things from patients' perspectives (reversed)			.709			.722

Eigenvalue	4.2	1.5	1.3	3.40	2.63	1.69

% of variance	21%	8%	7%	17.0%	13.1%	8.4%

*Printed with express permission from Dr. M. Hojat.

a: F1 = Perspective Taking, F2 = Compassionate Care, F3 = Standing in the Patient's Shoes.

b: F1 = Emotional Engagement/Compassionate Care, F2 = Perspective Taking, F3 = Standing in the Patient's Shoes.
